# Clinical outcomes of CD3xCD20 bispecific antibodies in patients with central nervous system involvement by lymphoma

**DOI:** 10.1038/s41408-026-01519-6

**Published:** 2026-05-22

**Authors:** Karan L. Chohan, Rina L. Welkie, James Godfrey, Steven Bair, Lorenzo Falchi, Manali Kamdar, Ajay Major, Wendy Dixon, Jennifer Crombie, Reid Merryman, Sonia Godbole, Gilles Salles, Omnia Farahat, Amy Ayers, Ayushi Chauhan, Alex Herrera, Geoffrey Shouse, Sairah Ahmed, Yazeed Sawalha

**Affiliations:** 1 Department of Lymphoma & Myeloma, The University of Texas MD Anderson Cancer Center, Houston, TX USA; 2 Division of Hematology, Department of Internal Medicine, Arthur G. James Comprehensive Cancer Center, The Ohio State University Wexner Medical Center, Columbus, OH USA; 3 City of Hope, Hematology and Hematopoietic Cell Transplantation, Duarte, CA USA; 4 Division of Hematology, University of Colorado—Anschutz Medical Campus, Aurora, CO USA; 5 Memorial Sloan Kettering Cancer Center, New York, NY USA; 6 Dana-Farber Cancer Institute, Boston, MA USA

**Keywords:** B-cell lymphoma, Cancer immunotherapy

Dear Editor,

Despite recent advances in the treatment of systemic aggressive B-cell non-Hodgkin lymphomas (B-NHL), central nervous system (CNS) involvement remains a therapeutic challenge and is associated with dismal outcomes [1]. Chimeric antigen receptor (CAR) T-cell therapy achieves high overall response rates (ORR) in patients with CNS involvement, but responses are often short-lived (1-year progression-free survival (PFS) of 16–48%) [2–5]. The CD3xCD20 bispecific antibodies (BsAbs) demonstrated high efficacy and a favorable safety profile in relapsed/refractory aggressive B-NHL; however, patients with active CNS involvement were excluded from the pivotal trials [6–8]. The data currently available on BsAb activity in patients with CNS lymphoma involvement are limited to a few small case series that suggest potential activity [9–11]. Moreover, the safety profile of BsAbs in patients with a prior history of CNS involvement but without active disease at the time of treatment initiation remains incompletely characterized, as this population has also been largely excluded from prospective studies [6, 7]. Herein, we report multicenter outcomes of CD3xCD20 BsAbs in patients with active or prior history of CNS involvement by aggressive B-NHL from the Collaborative US Bispecific Consortium (CUBIC).

This is a multicenter retrospective study of adult patients with active or a history of CNS involvement by an aggressive B-NHL treated with commercial CD3xCD20 BsAbs between 8/2023 and 7/2025 at six US medical centers as part of CUBIC. CNS involvement was defined as parenchymal, cerebrospinal fluid (CSF), and/or meningeal involvement, confirmed by biopsy, CSF cytology and/or flow cytometry, and/or brain/spine magnetic resonance imaging (MRI). The active CNS involvement cohort included patients with documented CNS disease at the time of BsAb treatment initiation, whereas the prior CNS involvement cohort included those with a history of CNS disease but no documented involvement at the time of BsAb initiation. Baseline CNS evaluation, including brain and spine MRI and/or CSF analysis, was at the treating physician’s discretion. Response to treatment was determined by the treating physician using PET/CT for systemic involvement (per Lugano criteria) and brain/spine MRI and/or CSF analysis for CNS involvement. Toxicity grading for cytokine release syndrome (CRS) and immune effector cell neurotoxicity syndrome (ICANS) was per the ASTCT Consensus Grading criteria [12, 13]. We used descriptive statistics to summarize patient characteristics, and the Kaplan-Meier method to estimate survival. PFS was calculated from BsAb initiation to disease progression (PD) (systemic or CNS) or death, CNS-specific PFS from BsAb initiation to CNS PD, and overall survival (OS) from BsAb initiation to death. This study received approval from the institutional review boards of all involved sites.

Twenty-eight patients with relapsed/refractory aggressive B-NHL received BsAb therapy (epcoritamab, *n* = 5; glofitamab, *n* = 22; mosunetuzumab, *n* = 1) for active (*n* = 18) or prior CNS involvement (*n* = 10). Baseline characteristics are presented in Supplementaty Table 1.

In patients with active CNS involvement (*n* = 18), the median age at treatment was 68 years (range, 40-82), 61% were male, and 59% had an ECOG performance status (PS) ≥ 2. Thirteen patients (72%) had DLBCL, 2 (11%) high-grade B cell lymphoma (HGBL), 2 (11%) primary CNS lymphoma (PCNSL), and 1 (6%) mantle cell lymphoma (MCL). The median time from lymphoma diagnosis to first known CNS involvement (excluding PCNSL patients) was 7 months (range, 0–94). The median time from first CNS involvement to BsAb treatment was 8 months (range, 0–64) for patients with secondary CNS involvement, and 9 and 14 months for the two patients with PCNSL. Seven patients (39%) started BsAb within 3 months of first CNS involvement. Thirteen patients (72%) had synchronous CNS and systemic disease, and 5 (28%) had isolated CNS disease. Twelve patients (67%) had parenchymal disease, 3 (17%) had leptomeningeal disease (LMD) including 1 with additional vitreoretinal involvement, and 3 (17%) had parenchymal and LMD. Prior to BsAb, patients received a median of 3 lines of therapy (range, 2–9), including CAR T-cell therapy in 11 patients (61%), with a median of 10 months (range, 1–48) prior to BsAb initiation. Fourteen patients (78%) were refractory to the last prior line of therapy. Fifteen patients received glofitamab, 2 epcoritamab, and 1 mosunetuzumab. Twelve patients (67%) received the BsAb in combination: a BTK inhibitor (*n* = 4, acalabrutinib or zanubrutinib), polatuzumab vedotin (*n* = 1), lenalidomide (*n* = 1), systemic chemotherapy (*n* = 4), IT chemotherapy (*n* = 2), or CNS-directed RT (n = 3).

The best ORR (systemic + CNS) was 56% (10/18), including CR in 33% (6/18). The best CNS ORR was 65% (11/17; 1 patient was unevaluable for CNS response but had systemic PD), including CR in 35% (6/17). The median time to best CNS response was 58 days (range, 19–364). Table 1 lists the overall (systemic and CNS) and CNS ORR and CR rates in different subgroups. Among the 6 patients who achieved CNS CR, 3 had concurrent systemic disease and achieved systemic CR. With a median follow-up of 12 months (range, 4–21) from BsAb initiation, none of these 6 patients developed CNS or systemic relapse or received subsequent treatments.

**Table 1:** Best systemic and CNS response rates among patients with active CNS lymphoma.

Variable	(CNS + systemic)		CNS^a^	
	ORR, *n* (%)	CR, *n* (%)	ORR, *n* (%)	CR, *n* (%)
All patients, *n* = 18^a^	10 (56%)	6 (33%)	11 (65%)	6 (35%)
Bispecific agent				
▪ Glofitamab, *n* = 15^a^	9 (60%)	5 (33%)	10 (71%)	5 (36%)
▪ Epcoritamab, *n* = 2	1 (50%)	1 (50%)	1 (50%)	1 (50%)
▪ Mosunetuzumab, *n* = 1	0	0	0	0
CNS site				
▪ Parenchymal, *n* = 12	8 (67%)	5 (42%)	9 (75%)	5 (42%)
▪ Leptomeningeal, *n* = 3	2 (67%)	1 (33%)	2 (67%)	1 (33%)
▪ Both, *n* = 3^a^	0	0	0	0
Treatment type				
▪ Monotherapy, *n* = 6^a^	4 (67%)	3 (50%)	4 (80%)	3 (60%)
▪ Combination, *n* = 12	6 (50%)	3 (25%)	7 (58%)	3 (33%)
Lymphoma histology				
▪ DLBCL, *n* = 13^a^	8 (62%)	5 (38%)	9 (75%)	5 (42%)
▪ HGBL, *n* = 2	0	0	0	0
▪ PCNSL, *n* = 2	1 (50%)	1 (50%)	1 (50%)	1 (50%)
▪ MCL, *n* = 1	1 (100%)	0	1 (100%)	0
Prior CAR T-cell therapy				
▪ Yes, *n* = 11	7 (64%)	4 (36%)	8 (73%)	4 (36%)
▪ No, *n* = 7^a^	3 (43%)	2 (29%)	3 (50%)	2 (33%)
Time from CART to bispecific				
▪ ≤6 months, *n* = 4	2 (50%)	2 (50%)	2 (50%)	2 (50%)
▪ >6 months, *n* = 7	5 (71%)	2 (29%)	6 (86%)	2 (29%)
Time from first CNS involvement to bispecific				
▪ ≤6 months, *n* = 8^a^	5 (63%)	3 (38%)	5 (71%)	3 (43%)
▪ >6 months, *n* = 10	5 (50%)	3 (30%)	6 (60%)	3 (30%)
Prior lines of treatment				
▪ 2, *n* = 9^a^	6 (67%)	4 (44%)	6 (75%)	4 (50%)
▪ ≥3, *n* = 9	4 (44%)	2 (22%)	5 (56%)	2 (22%)

^a^1 patient was unevaluable for CNS response but had systemic PD.

*CAR T* chimeric antigen receptor T-cell therapy, *CNS* central nervous system, *CR* complete response, *DLBCL* diffuse large B-cell lymphoma, *HGBL* high-grade B-cell lymphoma, *MCL* mantle cell lymphoma, *ORR* overall response rate, *PCNSL* primary central nervous system lymphoma, *PD* progressive disease.

With a median follow-up of 6 months (95% confidence interval (CI), 4–14), the 6-month PFS and OS were 43% (95% CI, 20–64%) and 77% (95% CI, 49–91%), respectively (Fig. 1A, B). The 6-month CNS PFS and systemic PFS were 53% (95% CI, 27–73%) and 56% (95% CI, 28–77%). Of the 9 patients with PD, 4 had CNS PD and 6 had systemic PD (one patient had both). The 6-month PFS (Fig. 1C) and CNS-specific (Fig. 1D) did not significantly differ between monotherapy (50% and 67%, respectively) and combination therapy (38% and 43%, respectively) (*p* = 0.66 and 0.44, respectively).

**Fig. 1:**
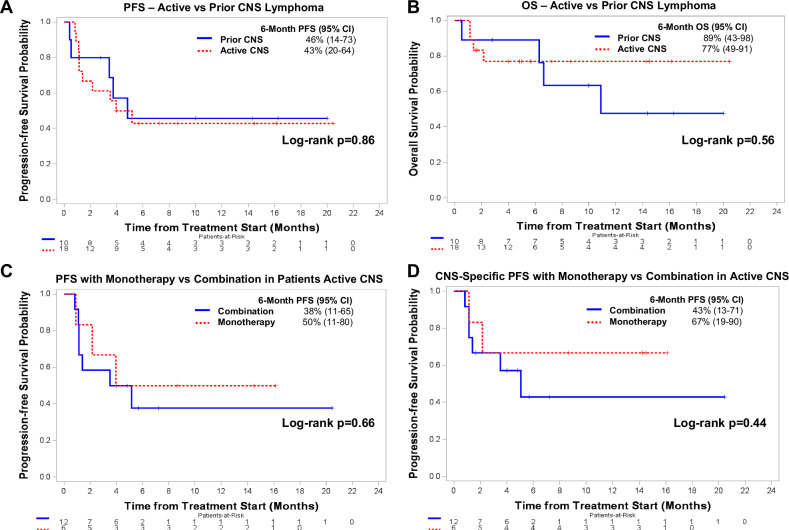
Progression-free survival (PFS) and overall survival (OS) curves stratified by CNS involvement and treatment strategy. PFS and OS based on active or prior history of CNS involvement in all patients (**A**, **B**). PFS and CNS-Specific PFS based on combination or monotherapy treatment strategy for patients with active CNS disease (**C**, **D**).

In patients with prior CNS involvement (*n* = 10), the median age at treatment was 63 years (range, 22–82), 70% were male, and 50% had an ECOG PS ≥ 2. Five patients (50%) had DLBCL, 2 HGBL (20%), and 1 of each had PCNSL, MCL, and Burkitt lymphoma. The median time from first CNS involvement to BsAb treatment initiation was 9 months (range, 4–79). Prior to BsAb, patients received a median of 2 lines of therapy (range, 2–4), including CAR T-cell therapy in 7 patients (70%) and CNS RT in 3 (30%). Seven patients received glofitamab and 3 epcoritamab. Two patients (20%) received BsAb in combination with polatuzumab vedotin and one with CNS-directed RT.

The ORR was 60% (6/10), including CR in 50% (5/10). With a median follow-up of 14 months (95% CI, 0.5-not reached), the 6-month PFS and OS rates were 46% and 89%, respectively. During follow-up, 2 patients (20%) developed CNS relapse concurrently with systemic PD.

Among all patients, CRS occurred in 12 patients (43%), including grade 1 in 8 (29%) and grade 2 in 4 (14%). ICANS occurred in 7 patients (25%), with grade 1 in 4 (14%) and grades 2, 3, and 4 in 1 patient (4%) each. Of these 7 patients with ICANS, 4 had DLBCL, 2 MCL, and 1 HGBL; 6 received glofitamab and 1 epcoritamab; 5 received the BsAb in combination (systemic chemotherapy, *n* = 3; IT chemotherapy, n = 1; or RT, *n* = 1); 4 did not develop CRS; 4 had active CNS lymphoma (2 parenchymal, 2 LMD), and 3 had prior CNS involvement. None of the 4 patients with active CNS lymphoma who developed ICANS achieved CR (3 achieved PR as best response).

Our study demonstrates significant CNS clinical activity for the CD3xCD20 BsAbs, as monotherapy or in combination, in aggressive B-NHL (CNS ORR of 65% including CR in 35%). Further, none of the 6 patients who achieved CR relapsed during a median follow-up of 12 months, although we acknowledge the small sample size and relatively short follow-up. Among patients with prior CNS involvement, CNS relapse occurred in 2 of 10 patients during follow-up; however, the limited size of this subgroup precludes definitive conclusions regarding treatment effect. While the CRS rates in our study were comparable to those in the pivotal BsAb trials and real-world studies, we observed higher ICANS rates, including in patients with prior CNS involvement.

Other smaller retrospective studies reported on the activity of BsAbs in patients with CNS involvement [9–11]. A report of 4 patients treated with glofitamab showed responses in 3, none of whom developed ICANS [10]. Correlative analyses from this study demonstrated that glofitamab is detectable in CSF samples, and there is increased immune infiltration in the CSF following BsAb treatment [10]. A study showed responses to glofitamab in 5 of 6 response-evaluable patients with secondary CNS involvement by B-NHL, including CR in 3 (1 patient developed ICANS), whereas another study did not show responses in 5 evaluable patients treated with epcoritamab or glofitamab [9, 14]. A report of 3 patients with relapsed/refractory Burkitt lymphoma treated with glofitamab-polatuzumab showed CR in the patient with CNS involvement [15].

The limitations of this study include its retrospective design, limited sample size, short follow-up, heterogeneous treatment strategies, and inclusion of various lymphoma subtypes. Additionally, the subgroup of patients with prior CNS involvement was small, limiting conclusions regarding safety or efficacy in this population.

To our knowledge, this is the largest real-world analysis evaluating the use of CD3×CD20 BsAbs in patients with CNS involvement by aggressive B-NHL. This patient population is underrepresented in clinical trials, and prospective studies will be needed to better define the role of these agents in this setting. Further, ICANS rates appeared higher in our cohort, emphasizing the importance of careful monitoring in patients with active CNS disease or a history of CNS involvement. Notably, durable responses were observed in a subset of patients treated with these agents.

## Supplementary information

Supplemental File

## Data Availability

Please contact the corresponding author to request clinical data.
