# Supplementary material for: Clinical outcomes of CD3xCD20 bispecific antibodies in patients with central nervous system involvement by lymphoma
Source: Blood Cancer J. 2026 May 22;16(1):81. doi: 10.1038/s41408-026-01519-6 (PMC13197401; doi:10.1038/s41408-026-01519-6)
Supplement: Supplementary file 1 — Supplemental File [file 41408_2026_1519_MOESM1_ESM.pdf]

Supplemental Material

Title: Clinical Outcomes of CD3xCD20 Bispecific Antibodies in Patients with Central Nervous System Involvement by Lymphoma

Running Title: CD3xCD20 bispecific antibodies for CNS lymphoma

Karan L. Chohan[1], Rina L Welkie[2], James Godfrey[3], Steven Bair[4], Lorenzo Falchi[5], Manali Kamdar[4], Ajay Major[4], Wendy Dixon[6], Jennifer Crombie[6], Reid Merryman[6], Sonia Godbole[2], Gilles Salles[5], Omnia Farahat[1], Amy Ayers[1], Ayushi Chauhan[1], Alex Herrera[3], Geoffrey Shouse[3], Sairah Ahmed[1], Yazeed Sawalha[2]*

1 - Department of Lymphoma & Myeloma, The University of Texas MD Anderson Cancer Center, Houston, TX, United States
2 - Arthur G. James Comprehensive Cancer Center, The Ohio State University Wexner Medical Center, Department of Internal Medicine, Division of Hematology, Columbus, United States
3 - City of Hope, Hematology and Hematopoietic Cell Transplantation, Duarte, United States
4 - University of Colorado – Anschutz Medical Campus, Division of Hematology, Aurora, United States
5 - Memorial Sloan Kettering Cancer Center, New York City, United States
6 – Dana-Farber Cancer Institute, Boston, United States

***Corresponding Author:**

Yazeed Sawalha, MD

Email: yazeed.sawalha@osumc.edu

Supplemental Table 1: Baseline characteristics of all patients and stratified by active or prior history of CNS disease

Characteristics*	All Patients (n=28)	Prior CNS (n=10)	Active CNS (n=18)
Age at Bispecific, years, median (range)	68 (22, 82)	63 (22, 82)	68 (40, 82)
Male gender	18 (64.3)	7 (70.0)	11 (61.1)
ECOG Status			
0-1	12 (44.4)	5 (50.0)	7 (41.2)
2-4	15 (55.6)	5 (50.0)	10 (58.8)
Bispecific Agent			
Epcoritamab	5 (17.9)	3 (30.0)	2 (11.1)
Glofitamab	22 (78.6)	7 (70.0)	15 (83.3)
Mosunetuzumab	1 (3.6)	0 (0.0)	1 (5.6)
Histology,			
Burkitt Lymphoma	1 (3.6)	1 (10.0)	0 (0.0)
Diffuse Large B Cell Lymphoma	18 (64.3)	5 (50.0)	13 (72.2)
High-Grade B Cell Lymphoma	4 (14.3)	2 (20.0)	2 (11.1)
Mantle Cell Lymphoma	2 (7.1)	1 (10.0)	1 (5.6)
Primary CNS Lymphoma	3 (10.7)	1 (10.0)	2 (11.1)
LDH >ULN	17 (63.0)	7 (70.0)	10 (58.8)
Site of CNS involvement at BsAb administration			
Parenchymal-only	12 (42.9)	-	12 (66.7)
Leptomeningeal-only	3 (10.7)	-	3 (16.7)
Both	3 (10.7)	-	3 (16.7)
CNS Disease Status at BsAb administration			
Isolated CNS disease	5 (17.9)	-	5 (27.8)
Synchronous CNS and systemic involvement	13 (46.4)	-	13 (72.2)
Number of Prior Lines of Therapy, median (range)	2 (2, 9)	2 (2, 4)	3 (2, 9)
Prior CART, n (%)	18 (64.3)	7 (70.0)	11 (61.1)

*n(%), unless otherwise indicated. CNS, central nervous system; ECOG, Eastern Cooperative Oncology Group; LDH, lactate dehydrogenase; ULN, upper limit of normal; BsAb, bispecific antibody; CAR T, chimeric antigen receptor T cell.
